# Retinal oxygen extraction in humans

**DOI:** 10.1038/srep15763

**Published:** 2015-10-27

**Authors:** René M. Werkmeister, Doreen Schmidl, Gerold Aschinger, Veronika Doblhoff-Dier, Stefan Palkovits, Magdalena Wirth, Gerhard Garhöfer, Robert A. Linsenmeier, Rainer A. Leitgeb, Leopold Schmetterer

**Affiliations:** 1 Center for Medical Physics and Biomedical Engineering, Medical University of Vienna, Waehringer Guertel 18-20, 1090 Vienna, Austria; 2 Department of Clinical Pharmacology, Medical University of Vienna, Waehringer Guertel 18-20, 1090 Vienna, Austria; 3 Institute of Applied Physics, Vienna University of Technology, Wiedner Hauptstr, 8-10, 1040 Vienna, Austria; 4 Department of Biomedical Engineering, Northwestern University, Evanston, IL 60208, USA; 5 Department of Ophthalmology, Northwestern University, Chicago, IL 60611, USA; 6 Department of Neurobiology and Physiology, Northwestern University, Evanston, IL 60208.

## Abstract

Adequate function of the retina is dependent on proper oxygen supply. In humans, the inner retina is oxygenated via the retinal circulation. We present a method to calculate total retinal oxygen extraction based on measurement of total retinal blood flow using dual-beam bidirectional Doppler optical coherence tomography and measurement of oxygen saturation by spectrophotometry. These measurements were done on 8 healthy subjects while breathing ambient room air and 100% oxygen. Total retinal blood flow was 44.3 ± 9.0 μl/min during baseline and decreased to 18.7 ± 4.2 μl/min during 100% oxygen breathing (P < 0.001) resulting in a pronounced decrease in retinal oxygen extraction from 2.33 ± 0.51 μl(O_2_)/min to 0.88 ± 0.14 μl(O_2_)/min during breathing of 100% oxygen. The method presented in this paper may have significant potential to study oxygen metabolism in hypoxic retinal diseases such as diabetic retinopathy.

In 2010, a total of 185 million people were legally blind worldwide^1^. In industrialized countries, the three major causes of blindness are age-related macular degeneration, glaucoma and diabetic retinopathy. Alterations in ocular blood flow and oxygenation have been implicated in all of these conditions^2^,^3^.

The human retina has a complex system of vascular supply to ensure adequate oxygenation. The inner retina including the retinal ganglion cells (RGC) is nourished by the retinal circulation whereas the outer retina is supplied by the choroidal vasculature^4^. As in the brain, the retina has a continuous demand for oxygen and, as such, a lack of oxygen delivery will be associated with hypoxia leading to retinal disease^5^,^6^.

Most of the knowledge about retinal oxygenation arises from animal studies using microelectrodes^7^,^8^,^9^. This technique allows for the mapping of oxygen gradients with high spatial and temporal resolution and retinal oxygen profiles in pigs^10^, cats^11^, rats^12^ and monkeys^13^,^14^ were published based on this method. Except for the monkey fovea, oxygen profiles in these species share a lot of similarities. Under light-adapted conditions, they are characterized by a steep fall between the choriocapillaris and the photoreceptor inner segments^5^. Subsequently, oxygen tension decreases more gradually through the outer retina and has a value of approximately 20 mmHg in the inner retina^5^.

In humans, measurement of oxygen saturation with microelectrodes is not applicable due to the invasive nature of this technique. Here we describe a new method for the measurement of total retinal oxygen extraction in healthy subjects. This was done by combining measurements of total retinal blood flow using dual-beam bidirectional Doppler Fourier domain optical coherence tomography^15^ (FD-OCT) with measurements of oxygen saturation in retinal vessels using fundus reflectometry^16^,^17^. A mathematical model was developed to translate these measurements into total retinal oxygen extraction. Measurements were done during both breathing of ambient room air and during 100% oxygen breathing. During systemic hyperoxia, there is evidence that the amount of oxygen extracted from the retinal circulation shows a pronounced decrease, and we believe that this occurs because oxygen diffuses from the choroid to the inner retina^18^,^19^,^20^.

## Research Design and Methods

### Subjects

The study protocol was approved by the Ethics Committee of the Medical University of Vienna and followed the guidelines set forth in the Declaration of Helsinki. A total of eight healthy subjects (six male and two female) between 18 and 35 years were included in the study after signing written informed consent. All subjects passed a screening examination before the study day that included physical examination, blood draw to assess hematological status and chemistry, 12-lead electro cardiogram, assessment of visual acuity, slit lamp biomicroscopy, funduscopy and measurement of intraocular pressure (IOP). Exclusion criteria were ametropia ≥3 diopter, anisometropia ≥3 diopter, other ocular abnormalities and any clinically relevant illness as judged by the investigators, blood donation and/or intake of any medication in the three weeks prior to the study. Participants had to abstain from beverages containing alcohol or caffeine in the 12 hours before the study day.

### Protocol

After instillation of one drop of tropicamide (Mydriatikum AGEPHA, Vienna, Austria) into the study eye, a resting period of at least 20 minutes was scheduled. Thereafter, retinal blood velocities were measured using dual-beam bidirectional Doppler FD-OCT. Fundus photographs were taken using the Retinal Vessel Analyser (RVA, Imedos Systems UG, Jena, Germany) in order to quantify vessel diameters and oxygen saturation. A capillary blood sample was obtained from the earlobe for blood gas analysis. Blood pressure and pulse rate were measured non-invasively. Oxygen (gases for human use, Messer; Vienna, Austria) was provided via a partially expanded reservoir bag at atmospheric pressure. For gas delivery to the subject, a face mask which covered mouth and nose connected to a two-valve system, preventing the subject from rebreathing was used. After a resting period of 20 minutes, the phase of inhaling 100% oxygen that lasted 30 minutes began. Retinal hemodynamic measurements were performed 15 minutes after the start of inhalation.

### Measurement of retinal blood flow and oxygen saturation

The measurements were performed with a dual-beam Doppler FD-OCT system, described previously^15^,^21^,^22^. Two orthogonally polarized probe beams in the interferometer’s sample arm are focused onto the retina under a known angle Δ*α* between them. The system records the spectra of the two channels as a function of frequency using two identical spectrometers. When the probe beams fall onto moving red blood cells (RBC), the back reflected light in each channel is Doppler-shifted. These Doppler shifts Φ_1_ and Φ_2_ can be obtained by calculating the difference of phase of the Fourier transform of subsequent A-line recordings at the spectrometer’s CCD cameras which are operated at a line scan rate of *τ* =27 μs. Then, the phase difference ΔΦ=Φ_1_ − Φ_2_ between the two beams and system channels is calculated and the absolute flow velocity can be obtained as


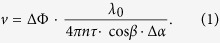


In equation (1), *λ*_0_=838.8 nm is the light source’s central wavelength, *n* = 1.37 is the refractive index of blood and *β* is the angle of the vessel’s blood flow velocity vector with respect to the plane spanned by the two probe beams. The angle Δ*α* was calculated for each subject’s eye individually based on the separation of the two probe beams at the pupil plane, the eye length and the ametropia value. All phase images were corrected for bulk-motion and phase wrapping^23^.

**Figure 1:**
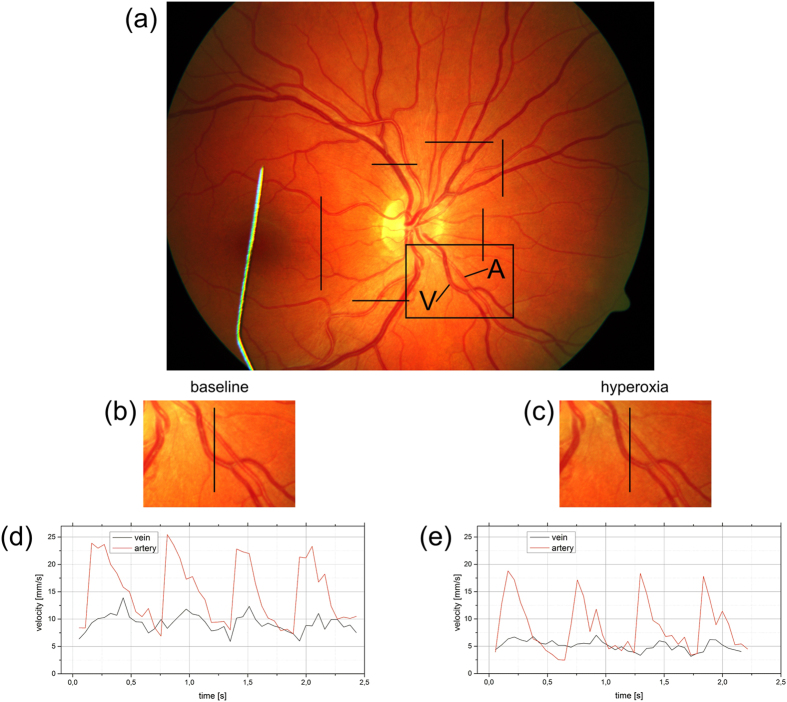
Sample measurement in a healthy subject. (**a**) Fundus image and OCT scanning patterns used to measure all retinal vessels entering the optic nerve head to measure total retinal blood flow. Black bars indicate the measurement locations. (**b**,**c**) Magnification of the fundus image during breathing room air and 100% oxygen, respectively. Vasoconstriction of retinal artery and vein can clearly be seen. (**d**,**e**) Time course of retinal arterial and venous blood velocity during breathing room air and breathing 100% oxygen respectively over approximately 4 cardiac cycles.

The OCT-setup was integrated into the RVA, which consists of a modified fundus camera (FF450plus; Carl Zeiss Meditec AG, Jena, Germany) and a CCD camera allowing for the measurement of retinal vessel diameters and oxygen saturation. The OCT beams are coupled into the RVA using a dichroic mirror, which ensures independent operation of the OCT system and the fundus camera. Using a rotatable beam displacer, the detection plane spanned by the two probe beams can either be aligned vertically or horizontally relative to the eye. This is required because without rotation of the detection plane, vessels with an orientation perpendicular to this plane would not be assessable, since the angle *β* in equation (1) would become zero. To determine the total retinal blood flow, OCT measurements are performed in a rectangular scanning pattern around the optic nerve head (ONH) (Fig. 1), which ensures that the mean velocities (*v*_A,i_, *v*_V,j_) in all the blood vessels leaving or entering the ONH can be measured. The measurement time per single location is approximately 5 s, which allows for averaging over several pulse periods. The angle *β* was determined from the fundus photographs recorded with the RVA’s camera.

Concomitantly with the OCT measurements, the diameters of the measured vessels (*d*_A,i_, *d*_V,j_) were determined with the RVA using static vessel analysis. The analyzing software was modified to allow for calculation of absolute values in μm based on the individual axial eye length, the axial refraction for each subject and the optical characteristics of the fundus camera system. Since the RVA measurements are less reliable for very small vessels, vessel diameters below 65 μm were measured in the OCT images and determined by comparison with the RVA results from larger vessels as described in detail previously^15^.

Using the mean velocity *v* from the OCT recordings and the vessel diameter *d* from the RVA measurements, blood flow *Q* in each individual vessel entering or leaving the ONH can be calculated as


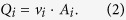


*A*_*i*_ is the cross sectional area of the vessel and is calculated assuming a circular cross section:


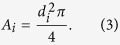


Due to the equation of continuity and because the retina is an end organ, the blood flow in the central vessels can be calculated based on these measurements. By summing up the blood flow values *Q*_A,i_ in all measured arteries, one can determine the blood flow *Q*_A,tot_ in the central retinal artery, and by summing up the blood flow values *Q*_V,j_ in all measured veins, it is possible to calculate the blood flow *Q*_V,tot_ in the central retinal vein. In formula (4) and formula (5), *#A* is the total number of measured arteries and *#V* is the total number of measured veins.


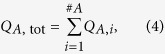



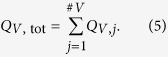


Obviously, *Q*_A,tot_ needs to be equal to *Q*_V,tot_, which provides a validity check for each measurement. Total retinal blood flow was calculated as the arithmetic mean of *Q*_A,tot_ and *Q*_V,tot_:


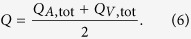


The fundus photographs were used to extract the oxygen saturation in retinal arteries and veins^16^,^24^. This is based on spectral analysis of light at selected wavelengths, which is reflected at the fundus. In the present system, two fundus pictures at wavelengths of 610 and 545 nm, respectively, are taken. Extraction of the retinal oxygen saturation is based on the fact that oxygenated hemoglobin has different light absorption characteristics as compared to deoxygenated hemoglobin. The isosbestic wavelength is 548 nm; it is defined as the point in the light spectrum where oxygenated and deoxygenated hemoglobin show identical absorption. On the other hand, oxygenated hemoglobin is nearly transparent if it is illuminated with light at a wavelength of 610 nm. Quantifying the contrast at these wavelengths enables the determination of the relation between oxygenated and total hemoglobin and the calculation of the oxygen saturation. The oxygen saturation was measured in all retinal arteries (*SaO*_2,A_)_*i*_ and all retinal veins *(SaO*_*2,V*_)_*j*_.

### Model to calculate total retinal oxygen extraction

**Figure 2:**
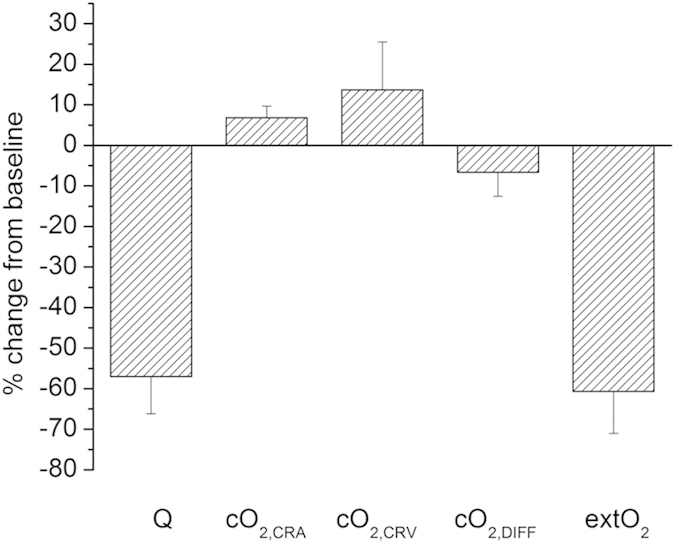
% change in total retinal blood flow (Q), arterial oxygen content (cO_2,CRA_), venous oxygen content (cO_2,CRV_), arteriovenous difference in oxygen content (cO_2,DIFF_) and retinal oxygen extraction (extO_2_) in response to 100% oxygen breathing (n = 8). Data are presented as means ± SD.

Measurements as described in the previous section were used to calculate the total retinal oxygen extraction. The visible arterial branch arteries at the posterior pole of the eye arise from the central retinal artery, which bifurcates at the center of the optic disk. The visible retinal branch veins merge into the central retinal vein. When measuring at some distance from the ONH, it is therefore necessary to correct the oxygen saturation readings for the oxygen loss across the arterial or venous wall between the point of measurement and the point where the vessels merge. The oxygen loss is described in formula (7) and depends on the diameter and the length of the vessel^25^:


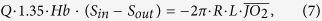


where *Q* is blood flow, *Hb* is the hemoglobin concentration, *S*_in_ − *S*_out_ is the difference in oxygen saturation in the specific vessel between the bifurcation point at the disk and the point where saturation is measured, *R* is the radius of the vessel, *L* is the distance between the point of measurement and the center of the ONH and 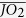 is the oxygen loss through the vascular wall. The value 1.35 is the amount of oxygen in [ml] which can bind to 1 g hemoglobin (Fig. 2).

Obviously, the oxygen loss across the wall depends on how many oxygen molecules diffuse into the vitreous or the retinal tissue. This value is described as the oxygen flux 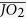. Measurements in the illuminated cat retina have shown that 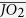 is 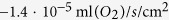. In the present model, this value was used for the calculation. To calculate the corrected oxygen saturation (*cSaO*_2_) when a vein leaves the retina, the estimated difference (*S*_in_ − *S*_out_) in the oxygen saturation has to be subtracted from the measured value (*SaO*_2_). In the arteries, on the other hand, the saturation at a point *L* from the ONH will be lower than the actual saturation of the central retinal artery, so the corrected value when the vessel joins the central retinal artery is higher than the measured value. Thus,


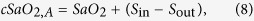



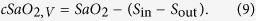


The value *cSaO*_2_ is used for the further calculations. The corrected oxygen saturations for all arteries in the posterior pole of the eye are used to calculate the mean value of the oxygen saturation in the central retinal artery (*SaO*_2,CRA_):


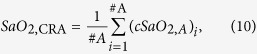


where *#A* is the total number of measured arteries.

The calculation of the oxygen saturation in the central retinal vein is more difficult, as the blood in the central retinal vein is a mixture of all veins in the back of the eye, which have different oxygen saturations. The corrected venous values in each vein also need to be weighted by the blood flow in each vein in order to calculate the oxygen saturation in the central retinal vein. The corrected oxygen saturation of each vein (*SaO*_2,V_)_*j*_ must be multiplied with the blood flow *Q*_V,j_ in the specific vessel and divided by the total flow in all veins *Q*_V,tot_ to obtain the central retinal vein’s oxygen saturation:


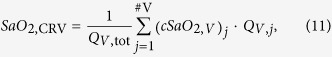


where *#V* is the number of measured retinal veins.

To calculate the retinal oxygen extraction of the human retina, we need to evaluate the amount of oxygen in the retinal vessels. The amount of oxygen includes two components: the amount bound to hemoglobin, and the amount dissolved in plasma. The amount of oxygen which is bound to hemoglobin can be easily calculated using the oxygen saturation, the hemoglobin concentration in the blood and the factor 1.35, as 1.35 ml oxygen is bound to 1 g hemoglobin. The fraction of dissolved oxygen can only be estimated, since it is not possible to measure the oxygen partial pressure in retinal vessels in humans. The oxygen partial pressure has to be multiplied by 0.003 ml O_2_/mmHg^26^. Under physiological conditions, the amount of oxygen that is dissolved is low. During 100% oxygen breathing, dissolved oxygen becomes, however, a significant proportion of the bloods oxygen content^27^.

To evaluate the arterial oxygen partial pressure, we used an arterialized blood sample from the earlobe. Venous PO_2_ was estimated from the saturation values by using the oxygen-binding curve^28^ (P50: 26.8 mmHg) at a PCO_2_ of 37 mmHg and a temperature of 37 degrees. Using these corrections, the oxygen content in the retinal vessels can finally be calculated as follows:


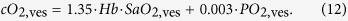


In this equation, *Hb* is the hemoglobin concentration in g per ml, 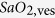 is the oxygen saturation and 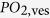 is the oxygen partial pressure; *ves* stands for the type of vessel (artery or vein).

The oxygen content of a vessel indicates how much oxygen is transported in a certain period of time. The oxygen extraction is the amount of oxygen which is consumed by the retinal tissues; it is calculated by subtracting the venous from the arterial oxygen content and multiplying by the blood flow, as given in formula (13).


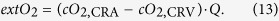


This formula was used to estimate total oxygen extraction from the retinal circulation. To obtain data per 100 g tissue, a value of 326 mg was chosen as total weight of the human retina^29^.

### Measurement of IOP and systemic hemodynamics

Systolic, diastolic and mean arterial blood pressures (SBP, DBP, MAP) were measured on the upper arm by an automated oscillometric device (Infinity Delta, Dräger, Vienna, Austria). The same device was used to record pulse rate and systemic oxygen saturation by a finger pulse oximeter.

### Blood gas analysis

Arterialized capillary blood from the earlobe was collected from a lancet incision into a thin glass capillary tube. Arterial pH, PCO2, and PO2 were determined using an automatic blood gas analysis system (AVL 912; CO-Oxilite, Graz, Austriax). Hemoglobin concentration (Hb) was measured photometrically (Sysmex XE 500, Kobe, Japan).

### Statistical analysis

Data are presented as means ±SD. Paired t-tests were used to compare baseline values to values obtained during 100% oxygen breathing. A p-value < 0.05 was considered the level of significance.

## Results

Baseline IOP in healthy subjects was 16.8 ± 1.4 mmHg. Breathing 100% oxygen did not induce a significant effect on either blood pressure or HR (Table 1). Retinal blood flow under room air conditions was 44.3 ± 9.0 μl/min. On average, 11.0 ± 2.2 arteries and 8.1 ± 1.6 veins were measured. There was no significant difference between *Q*_A,tot_ (44.2 ± 10.6 μl/min) and *Q*_V,tot_ (44.4 ± 7.6 μl/min), the absolute value of the mean difference between the 2 values was 8.0 ± 4.5%. During air breathing, the mean value of 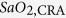 was 95.3 ± 1.9%, and the mean value of 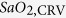
was 68.0 ± 3.9%. With corrected oxygen content values of 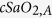 = 0.181 ± 0.008 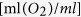 and 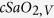 = 0.128 ± 0.011 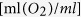, this resulted in a total retinal oxygen extraction (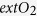) of 2.33 ± 0.51 μl(O_2_)/min. Analysis of both histological^30^ and OCT^31^ data for total and inner retinal thickness and numerical integration of these gave values of 317 mg and 137 mg for the total retinal mass and the mass of the inner retina, respectively (unpublished data). The weight of the retina was found to be in good agreement with the literature value of 326 mg^29^. This gives an inner retinal fraction of 0.43. However, the retinal circulation also supplies about 11 percent of the outer retina in macaque^14^, yielding that it provides blood to about half of the retinal weight. Taking these values into account, the result for retinal oxygen extraction can be written as 1.42 ± 0.31 ml(O_2_)/min/100g tissue.

The percent changes of retinal parameters during oxygen breathing are depicted in Fig. 2. During 100% oxygen breathing, we observed a pronounced decrease in retinal blood flow to a value of 18.7 ± 4.2 μl/min (p < 0.001). Again, there was no difference between *Q*_A,tot_ (19.0 ± 4.1 μl/min) and *Q*_V,tot_ (18.4 ± 4.5 μl/min), and the average absolute value of the difference was small (9.2 ± 3.8%). Both 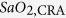 and 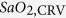 increased to values of 99.4 ± 0.3% (p = 0.004) and 76.6 ± 5.2% (p = 0.015), respectively. Hence, corrected oxygen saturation values during 100% oxygen breathing also increased (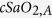 = 0.194 ± 0.010 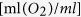, p < 0.001 and 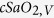 = 0.137 ± 0.013 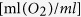 , p = 0.012). Since both retinal blood flow and the arterio-venous difference in corrected oxygen content decreased, we also observed a pronounced decrease in retinal oxygen extraction during 100% oxygen breathing (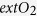 = 0.88 ± 0.14 μl(O_2_)/min or 0.54 ± 0.28 ml(O_2_)/min/100g tissue).

Table 2 shows data obtained in one healthy subject in all measured blood vessels. Figure 1 shows a sample measurement during room air breathing (b,d) and breathing 100% oxygen (c,e).

## Discussion

We present a method that is capable of non-invasively measuring total retinal oxygen extraction in humans. The value of 2.33 μl(O_2_)/min or 1.42 ml(O_2_)/min/100 g tissue blood is considerably lower than the value of 8 ml(O_2_)/min/100g tissue estimated previously^32^. The main difference is that the authors of this previous study estimated a flow rate of retinal blood as high as 170 ml/100 g tissue/min, which is much higher than the values obtained in the present study. Our values for retinal blood flow are, however, in the same range as those obtained by other authors using a variety of different methods^15^,^33^,^34^,^35^,^36^,^37^,^38^,^39^.

Our data are also lower than those obtained from animal experiments, but the difference is less pronounced. Under photopic conditions, several authors reported oxygen consumption values for the inner retina using different techniques. Employing Fick’s principle, a value of 3.9 ml(O_2_)/min/100 g tissue blood was reported in the pig retina^40^. Lower values of 2.3 ml(O_2_)/min/100 g tissue blood were reported in the rat by using the Warburg technique, an *in vitro* application of Fick’s law^41^. Values were also reported for inner retinal oxygen consumption in the cat^42^. By modeling data from microelectrode experiments during occlusion of the central retinal artery, the authors reported 3.9 ml(O_2_)/min/100 g tissue blood. It needs, however, to be considered that data from experiments using microelectrodes represent local values from the center of the retina. By contrast, our data as well as the data obtained via Fick’s method represent total retinal oxygen extraction including the peripheral retina, which contains less RGCs and less microvessels^4^,^43^.

Another study reported values for inner retinal oxygen extraction in the rat of 516 nl(O2)/min^44^. In this study, oxygen tension was measured by phosphorescence lifetime imaging and total retinal blood flow was measured by red-free and fluorescent microsphere imaging. In another recent study in rats, the inner retinal oxygen extraction was reported to be 300 nl(O_2_)/min^45^. In this study, total retinal blood flow was measured via Doppler FD-OCT, which is feasible as in anesthetized animals it is, due to the absence of sample motion, not required to use a dual-beam system that is insensitive to changes in the Doppler angle. Photoacoustic ophthalmoscopy was used to measure oxygen saturation. Using literature data for the weight of the rat’s retina of 14 mg^46^ and considering that 50% of oxygen consumption takes place in the inner retina this can be converted to 7.4 ml(O_2_)/min/100 g tissue (39) and 4.3 ml(O_2_)/min/100 g tissue (40) for the two studies, respectively.

Our data indicate that during 100% oxygen breathing there is a pronounced decrease in oxygen extraction from the retinal circulation. This is caused by a pronounced decrease in retinal blood flow as well as a decrease in arterio-venous oxygen difference. Our results are in good agreement with our previous data in which oxygen extraction was estimated from measurements on single arteries and veins^20^. In animal studies, it has been shown that inner retinal oxygen tension is well regulated during 100% oxygen breathing^18^,^19^,^47^,^48^. In these experiments, delivery of oxygen by the retinal circulation shows a pronounced decrease due to a pronounced decrease in retinal blood flow. On the other hand, choroidal blood flow shows almost no reaction to systemic hyperoxia^49^, which leads to significant diffusion of oxygen from the choroid to the inner retina.

The results of the present study are critically dependent on the validity of the underlying techniques. Measurement of retinal vessel diameters based on fundus photography is the gold standard technique providing excellent reproducibility and sensitivity^50^. The validity of dual-beam bidirectional Doppler FD-OCT is shown by several previous experiments. There is a high degree of association with blood velocities as measured with laser Doppler velocimetry, a technique widely used in clinical research, the law of mass conservation is well fulfilled at retinal bifurcations, and Murray’s law is fulfilled in our measurements^15^,^22^,^23^. In addition, values of total retinal arterial flow and total retinal venous flow are in good agreement in this study as it was the case in our previous experiments^15^, as can be expected for an end organ.

Measurements of oxygen saturation in retinal vessels were based on a two wavelength approach of fundus reflectometry. This technique, which is based on the different absorption characteristics of oxy- and deoxy-hemoglobin has several limitations. When photons enter the camera, the optical pathway is generally unknown. Hence, not all photons arising from the locations of the blood vessels have necessarily travelled through the entire vessel due to scattering. In addition, scattered photons from the areas adjacent to the vessel may have undergone absorption in the melanin of the retinal pigment epithelium. As such it does not come as a surprise that Monte Carlo simulation indicates an influence of vessel diameter and melanin on the accuracy of oxygen saturation measurements^51^. In the present system, this is overcome by compensating for these parameters based on a large dataset of normal values^16^. Validity of data as obtained with two wavelength systems as employed in the present study is strongly dependent on this calibration process. With the device produced by Imedos this calibration is based on retinal vessel reflectance spectra with a 2-nm resolution^52^. Nevertheless other investigators reported lower values for SaO_2,V_ in healthy volunteers with a mean value of 60%^53^. This has an effect on our calculation of oxygen extraction based on equation (13) and could lead to a slight underestimation of values with our technology. On the other hand, experimental evidence indicates that reduced arterial oxygen values in patients with Eisenmenger’s syndrome^54^, chronic obstructive pulmonary disease^55^ and breathing gas mixtures with reduced fraction of inspired oxygen^56^ are well reflected in the retinal oxygen saturation measurements. Hence, the difference between 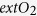 values during normoxia and hyperoxia are not affected by this limitation.

In conclusion, we present a system that is capable of measuring retinal oxygen extraction in humans. This technique may have considerable potential for diagnosis, and risk stratification treatment monitoring in patients with retinal vascular disease.

## Additional Information

**How to cite this article**: Werkmeister, R. M. *et al*. Retinal oxygen extraction in humans. *Sci. Rep*. **5**, 15763; doi: 10.1038/srep15763 (2015).

## Figures and Tables

**Table 1:** Blood pressure and pulse rate at baseline and during 100% oxygen breathing (n = 8). Data are presented as means ± SD.

	Baseline	100% oxygen breathing	p-value
Systolic blood pressure (mmHg)	119.5 ± 6.3	121.6 ± 7.1	0.48
Diastolic blood pressure (mmHg)	69.8 ± 11.9	72.8 ± 7.7	0.41
Mean blood pressure (mmHg)	87.9 ± 7.4	91.8 ± 5.0	0.19
Heart rate (beats/min)	69.1 ± 16.4	66.1 ± 9.8	0.33

**Table 2:** Values as obtained in one healthy subject in all retinal vessels. (I = inferior, N = nasal, S = superior, T = temporal).

Vessel	Artery(A)/ Vein(V)	Velocity (mm/s)	Diameter (μm)	Flow (μl/min)	Oxygen saturation (%)	Oxygen content (ml(O_2_)/ml)
I2	A	19.48	111.6	10.23	93.7	0.941
I3	A	7.53	70.2	1.56	93.2	0.950
I5	A	10.26	79.1	2.71	93.5	0.946
N1	A	7.94	80.8	2.19	92.4	0.938
N3	A	13.45	79.7	3.60	91.9	0.928
S2	A	7.78	65.6	1.41	77.8	0.796
S4	A	10.70	91.1	3.74	91.2	0.922
T2	A	10.22	82.1	3.14	94.3	0.953
T5	A	9.38	83.3	2.74	92.6	0.938
T7	A	15.79	109.1	7.93	91.5	0.920
I1	V	12.83	135.7	9.96	62.7	0.622
I4	V	5.34	93.5	1.97	67.2	0.653
I6	V	5.69	107.2	2.76	55.5	0.540
N2	V	6.31	108.6	3.14	59	0.576
N4	V	7.02	109.5	3.55	71.44	0.702
S1	V	8.40	165.5	9.71	67.8	0.671
S3	V	4.64	103.1	2.08	65.6	0.637
T1	V	2.72	80.7	0.75	57.1	0.529
T3	V	1.96	55	0.88	63.4	0.610
T4	V	7.87	71.1	1.68	62.4	0.607
T6	V	7.53	95.9	2.92	59.2	0.579

## References

[b1] Pascolini D. & Mariotti S. P. Global estimates of visual impairment: 2010. Br J Ophthalmol 96, 614–618, 10.1136/bjophthalmol-2011-300539 (2012). 22133988

[b2] Pemp B. & Schmetterer L. Ocular blood flow in diabetes and age-related macular degeneration. Can J Ophthalmol 43, 295–301, 10.3129/i08-049 (2008). 18443612

[b3] Cherecheanu A. P., Garhofer G., Schmidl D., Werkmeister R. & Schmetterer L. Ocular perfusion pressure and ocular blood flow in glaucoma. Curr Opin Pharmacol 13, 36–42, 10.1016/j.coph.2012.09.003 (2013). 23009741 PMC3553552

[b4] Pournaras C. J., Rungger-Brandle E., Riva C. E., Hardarson S. H. & Stefansson E. Regulation of retinal blood flow in health and disease. Prog Retin Eye Res 27, 284–330, 10.1016/j.preteyeres.2008.02.002 (2008). 18448380

[b5] Wangsa-Wirawan N. D. & Linsenmeier R. A. Retinal oxygen: fundamental and clinical aspects. Arch Ophthalmol 121, 547–557, 10.1001/archopht.121.4.547 (2003). 12695252

[b6] Cringle S. J. & Yu D. Y. Oxygen supply and consumption in the retina: implications for studies of retinopathy of prematurity. Doc Ophthalmol 120, 99–109, 10.1007/s10633-009-9197-2 (2010). 19830466

[b7] Whalen W. J., Riley J. & Nair P. A microelectrode for measuring intracellular PO2. J Appl Physiol 23, 798–801 (1967). 6061398 10.1152/jappl.1967.23.5.798

[b8] Tsacopoulos M. & Lehmenkuhler A. A double-barrelled Pt-microelectrode for simultaneous measurement of PO2 and bioelectrical activity in excitable tissues. Experientia 33, 1337–1338 (1977). 908408 10.1007/BF01920167

[b9] Linsenmeier R. A. & Yancey C. M. Improved fabrication of double-barreled recessed cathode O2 microelectrodes. Journal of applied physiology 63, 2554–2557 (1987). 3436887 10.1152/jappl.1987.63.6.2554

[b10] Pournaras C. J., Riva C. E., Tsacopoulos M. & Strommer K. Diffusion of O2 in the retina of anesthetized miniature pigs in normoxia and hyperoxia. Exp Eye Res 49, 347–360 (1989). 2792232 10.1016/0014-4835(89)90045-6

[b11] Alder V. A., Cringle S. J. & Constable I. J. The retinal oxygen profile in cats. Invest Ophthalmol Vis Sci 24, 30–36 (1983). 6826312

[b12] Cringle S. J., Yu D. Y. & Alder V. A. Intraretinal oxygen tension in the rat eye. Graefes Arch Clin Exp Ophthalmol 229, 574–577 (1991). 1765301 10.1007/BF00203324

[b13] Ahmed J., Braun R. D., Dunn R. Jr. & Linsenmeier R. A. Oxygen distribution in the macaque retina. Invest Ophthalmol Vis Sci 34, 516–521 (1993). 8449672

[b14] Birol G., Wang S., Budzynski E., Wangsa-Wirawan N. D. & Linsenmeier R. A. Oxygen distribution and consumption in the macaque retina. Am J Physiol Heart Circ Physiol 293, H1696–1704, 10.1152/ajpheart.00221.2007 (2007). 17557923

[b15] Doblhoff-Dier V. . Measurement of the total retinal blood flow using dual beam Fourier-domain Doppler optical coherence tomography with orthogonal detection planes. Biomed Opt Express 5, 630–642, 10.1364/BOE.5.000630 (2014). 24575355 PMC3920891

[b16] Hammer M., Vilser W., Riemer T. & Schweitzer D. Retinal vessel oximetry-calibration, compensation for vessel diameter and fundus pigmentation, and reproducibility. J Biomed Opt 13, 054015, 10.1117/1.2976032 (2008). 19021395

[b17] Geirsdottir A. . Retinal vessel oxygen saturation in healthy individuals. Invest Ophthalmol Vis Sci 53, 5433–5442, 10.1167/iovs.12-9912 (2012). 22786895

[b18] Chung C. K. & Linsenmeier R. A. Effect of carbogen (95% O2/5% CO2) on retinal oxygenation in dark-adapted anesthetized cats. Curr Eye Res 32, 699–707, 10.1080/02713680701459250 (2007). 17852195

[b19] Linsenmeier R. A. & Yancey C. M. Effects of hyperoxia on the oxygen distribution in the intact cat retina. Invest Ophthalmol Vis Sci 30, 612–618 (1989). 2703302

[b20] Palkovits S. . Retinal oxygen metabolism during normoxia and hyperoxia in healthy subjects. Invest Ophthalmol Vis Sci 55, 4707–4713, 10.1167/iovs.14-14593 (2014). 25015353

[b21] Werkmeister R. M. . Bidirectional Doppler Fourier-domain optical coherence tomography for measurement of absolute flow velocities in human retinal vessels. Opt Lett 33, 2967–2969 (2008). 19079508 10.1364/ol.33.002967

[b22] Werkmeister R. M. . Response of retinal blood flow to systemic hyperoxia as measured with dual-beam bidirectional Doppler Fourier-domain optical coherence tomography. PLoS One 7, e45876, 10.1371/journal.pone.0045876 (2012). 23029289 PMC3445512

[b23] Werkmeister R. M. . Measurement of absolute blood flow velocity and blood flow in the human retina by dual-beam bidirectional Doppler fourier-domain optical coherence tomography. Invest Ophthalmol Vis Sci 53, 6062–6071, 10.1167/iovs.12-9514 (2012). 22893675

[b24] Hammer M. . Diabetic patients with retinopathy show increased retinal venous oxygen saturation. Graefes Arch Clin Exp Ophthalmol 247, 1025–1030, 10.1007/s00417-009-1078-6 (2009). 19404666

[b25] Buerk D. G., Shonat R. D., Riva C. E. & Cranstoun S. D. O2 gradients and countercurrent exchange in the cat vitreous humor near retinal arterioles and venules. Microvasc Res 45, 134–148, 10.1006/mvre.1993.1013 (1993). 8361397

[b26] Nielsen H. B., Madsen P., Svendsen L. B., Roach R. C. & Secher N. H. The influence of PaO2, pH and SaO2 on maximal oxygen uptake. Acta physiologica Scandinavica 164, 89–87, 10.1046/j.1365-201X.1998.00405.x (1998). 9777029

[b27] Mason R. J. . Murray and Nadel’s Textbook of Respiratory Medicine. 2 edn, (Saunders W.B., 1994).

[b28] Leow M. K. Configuration of the hemoglobin oxygen dissociation curve demystified: a basic mathematical proof for medical and biological sciences undergraduates. Advances in physiology education 31, 198–201, 10.1152/advan.00012.2007 (2007). 17562911

[b29] Feke G. T. . Blood flow in the normal human retina. Invest Ophthalmol Vis Sci 30, 58–65 (1989). 2643588

[b30] Straatsma B. R., Foos R. Y. & Spencer L. M. in *In* New Orleans Academy of Ophthalmology Symposium on Retina and Retinal Surgery (St. Louis: CV Mosby, 1969).

[b31] Aleman T. S. . Inner retinal abnormalities in X-linked retinitis pigmentosa with RPGR mutations. Invest Ophthalmol Vis Sci 48, 4759–4765, 10.1167/iovs.07-0453 (2007). 17898302 PMC3178894

[b32] Hickam J. B. & Frayser R. Studies of Retinal Circulation in Man - Observations on Vessel Diameter Arteriovenous Oxygen Difference and Mean Circulation Time. Circulation 33, 302–& (1966). 25823104 10.1161/01.cir.33.2.302

[b33] Riva C. E., Grunwald J. E., Sinclair S. H. & Petrig B. L. Blood velocity and volumetric flow rate in human retinal vessels. Invest Ophthalmol Vis Sci 26, 1124–1132 (1985). 4019103

[b34] Grunwald J. E., Riva C. E., Baine J. & Brucker A. J. Total retinal volumetric blood flow rate in diabetic patients with poor glycemic control. Invest Ophthalmol Vis Sci 33, 356–363 (1992). 1740366

[b35] Polska E., Kircher K., Ehrlich P., Vecsei P. V. & Schmetterer L. RI in central retinal artery as assessed by CDI does not correspond to retinal vascular resistance. Am J Physiol Heart Circ Physiol 280, H1442–1447 (2001). 11247752 10.1152/ajpheart.2001.280.4.H1442

[b36] Wang Y., Bower B. A., Izatt J. A., Tan O. & Huang D. Retinal blood flow measurement by circumpapillary Fourier domain Doppler optical coherence tomography. J Biomed Opt 13, 064003, 10.1117/1.2998480 (2008). 19123650 PMC2840042

[b37] Baumann B. . Total retinal blood flow measurement with ultrahigh speed swept source/Fourier domain OCT. Biomed Opt Express 2, 1539–1552, 10.1364/BOE.2.001539 (2011). 21698017 PMC3114222

[b38] Garhofer G., Werkmeister R., Dragostinoff N. & Schmetterer L. Retinal blood flow in healthy young subjects. Invest Ophthalmol Vis Sci 53, 698–703, 10.1167/iovs.11-8624 (2012). 22247463

[b39] Sehi M. . Retinal blood flow in glaucomatous eyes with single-hemifield damage. Ophthalmology 121, 750–758, 10.1016/j.ophtha.2013.10.022 (2014). 24290800 PMC3943621

[b40] Wang L., Tornquist P. & Bill A. Glucose metabolism in pig outer retina in light and darkness. Acta physiologica Scandinavica 160, 75–81, 10.1046/j.1365-201X.1997.00030.x (1997). 9179314

[b41] Medrano C. J. & Fox D. A. Oxygen consumption in the rat outer and inner retina: light- and pharmacologically-induced inhibition. Exp Eye Res 61, 273–284 (1995). 7556491 10.1016/s0014-4835(05)80122-8

[b42] Braun R. D., Linsenmeier R. A. & Goldstick T. K. Oxygen consumption in the inner and outer retina of the cat. Invest Ophthalmol Vis Sci 36, 542–554 (1995). 7890485

[b43] Kur J., Newman E. A. & Chan-Ling T. Cellular and physiological mechanisms underlying blood flow regulation in the retina and choroid in health and disease. Prog Retin Eye Res 31, 377–406, 10.1016/j.preteyeres.2012.04.004 (2012). 22580107 PMC3418965

[b44] Wanek J., Teng P. Y., Blair N. P. & Shahidi M. Inner retinal oxygen delivery and metabolism under normoxia and hypoxia in rat. Invest Ophthalmol Vis Sci 54, 5012–5019, 10.1167/iovs.13-11887 (2013). 23821203 PMC3723378

[b45] Song W. . A combined method to quantify the retinal metabolic rate of oxygen using photoacoustic ophthalmoscopy and optical coherence tomography. Scientific Reports 4, 6525, 10.1038/srep06525 (2014). 25283870 PMC4185377

[b46] Blaszczyk W. M., Straub H. & Distler C. GABA content in the retina of pigmented and albino rats. Neuroreport 15, 1141–1144 (2004). 15129162 10.1097/00001756-200405190-00012

[b47] Riva C. E., Pournaras C. J. & Tsacopoulos M. Regulation of local oxygen tension and blood flow in the inner retina during hyperoxia. Journal of applied physiology 61, 592–598 (1986). 3745049 10.1152/jappl.1986.61.2.592

[b48] Yu D. Y., Cringle S. J. & Alder V. A. The response of rat vitreal oxygen tension to stepwise increases in inspired percentage oxygen. Invest Ophthalmol Vis Sci 31, 2493–2499 (1990). 2265989

[b49] Schmetterer L. . The effect of hyperoxia and hypercapnia on fundus pulsations in the macular and optic disc region in healthy young men. Exp Eye Res 61, 685–690 (1995). 8846840 10.1016/s0014-4835(05)80019-3

[b50] Garhofer G. . Use of the retinal vessel analyzer in ocular blood flow research. Acta Ophthalmol 88, 717–722, 10.1111/j.1755-3768.2009.01587.x (2010). 19681764

[b51] Liu W., Jiao S. & Zhang H. F. Accuracy of retinal oximetry: a Monte Carlo investigation. J Biomed Opt 18, 066003, 10.1117/1.JBO.18.6.066003 (2013). 23733019 PMC3669519

[b52] Hardarson S. H. & Stefansson E. Oxygen saturation in branch retinal vein occlusion. Acta Ophthalmol 90, 466–470, 10.1111/j.1755-3768.2011.02109.x (2012). 21518303

[b53] Hardarson S. H. . Automatic retinal oximetry. Invest Ophthalmol Vis Sci 47, 5011–5016, 10.1167/iovs.06-0039 (2006). 17065521

[b54] Traustason S. . Retinal oxygen saturation in patients with systemic hypoxemia. Invest Ophthalmol Vis Sci 52, 5064–5067, 10.1167/iovs.11-7275 (2011). 21467173

[b55] Palkovits S. . Measurement of retinal oxygen saturation in patients with chronic obstructive pulmonary disease. Invest Ophthalmol Vis Sci 54, 1008–1013, 10.1167/iovs.12-10504 (2013). 23307953

[b56] Palkovits S. . Regulation of retinal oxygen metabolism in humans during graded hypoxia. Am J Physiol Heart Circ Physiol 307, H1412–1418, 10.1152/ajpheart.00479.2014 (2014). 25217648

